# Measuring frailty in clinical practice: a comparison of physical frailty assessment methods in a geriatric out-patient clinic

**DOI:** 10.1186/s12877-017-0623-0

**Published:** 2017-11-13

**Authors:** J. M. Pritchard, C. C. Kennedy, S. Karampatos, G. Ioannidis, B. Misiaszek, S. Marr, C. Patterson, T. Woo, A. Papaioannou

**Affiliations:** 1 Geriatric Education and Research for the Aging Sciences (GERAS), St Peter’s Hospital, 88 Maplewood Ave, Hamilton, ON L8M 1W9 Canada; 2 Department of Kinesiology and Interdisciplinary Science, McMaster University, 1280 Main St West, Hamilton, ON L8S 4L8 Canada; 3 Department of Medicine, McMaster University, 1280 Main St West, Hamilton, ON L8S 4L8 Canada; 4 Population Health Research Institute (PHRI), St. Joseph’s Healthcare, Hamilton, ON L8N 4A6 Canada; 5 Department of Clinical Epidemiology and Biostatistics, McMaster University, 1280 Main St West, Hamilton, ON L8S 4L8 Canada

**Keywords:** Frailty, Phenotype, Fried, Short performance physical battery (SPPB), Geriatric medicine, Out-patient

## Abstract

**Background:**

The objectives of this study were to determine: 1) the prevalence of frailty using Fried’s phenotype method and the Short Performance Physical Battery (SPPB), 2) agreement between frailty assessment methods, 3) the feasibility of assessing frailty using Fried’s phenotype method and the SPPB.

**Methods:**

This cross-sectional study was conducted at a geriatric out-patient clinic in Hamilton, Canada. A research assistant conducted all frailty assessments. Patients were classified as non-frail, pre-frail or frail according to Fried’s phenotype method and the SPPB. Agreement among methods is reported using the Cohen kappa statistic (standard error). Feasibility data included the percent of eligible participants agreeing to attempt the frailty assessments (criterion for feasibility: ≥90% of patients agreeing to the frailty assessment), equipment required, and safety considerations. A *p*-value of <0.05 is considered significant.

**Results:**

A total of 110 participants (92%) and 109 participants (91%) agreed to attempt Fried’s phenotype method and SPPB, respectively. No adverse events occurred during any assessments. According to Fried’s phenotype method, the prevalence of frailty and pre-frailty was 35% and 56%, respectively, and according to the SPPB, the prevalence of frailty and pre-frailty was 50% and 35%, respectively. There was fair to moderate agreement between methods for determining which participants were frail (0.488 [0.082], *p* < 0.001) and pre-frail (0.272 [0.084], *p* = 0.002).

**Conclusions:**

Frailty and pre-frailty are common in this geriatric outpatient population, and there is fair to moderate agreement between Fried’s phenotype method and the SPPB. Over 90% of the patients who were eligible for the study agreed to attempt the frailty assessments, demonstrating that according to our feasibility criteria, frailty can be assessed in this patient population. Assessing frailty may help clinicians identify high-risk patients and tailor interventions based on baseline frailty characteristics.

## Background

Frailty is characterized by a loss of strength, endurance, physical ability and cognitive function, which results in an increased risk of vulnerability to disease, dependence, and death [1, 2]. Frail adults are at increased risk of falls, disability, hospitalization, admission to long-term care and mortality [3–5]. Independent of the number and severity of comorbidities measured by the Charlson Index, frail older adults who are hospitalized for an acute illness have a two-fold higher risk of mortality prior to discharge, compared to non-frail adults [5]. Healthcare spending is highest for frail older adults due to use of more expensive and intensive services [6], and adults over age 75 years with multiple comorbidities account for 60% of emergency room and hospital resource use [7]. These frail older adults with multiple comorbidities can be seen by geriatricians and supporting healthcare practitioners in geriatric medicine out-patient clinics based in acute care settings and in the community [8]. Screening patients in a clinical setting will help identify frail patients at high-risk for negative health outcomes and provide an opportunity to intervene and prevent the progression of frailty [9].

Although the negative consequences of frailty are well-established [3, 4], there is no gold standard method that is consistently used by researchers and clinicians to assess frailty. This may be because frailty is a multidimensional concept involving many physical, psychological and social aspects of health [1]. The Comprehensive Geriatric Assessment (CGA) is arguably the best way to assess overall health of an older adult, and the CGA is a multidisciplinary diagnostic procedure used to identify care needs and formulate future care plans for older adults [10]. However, conducting the CGA is resource intensive and does not objectively classify frail and non-frail patients by providing an overall frailty score. It may be advantageous for out-patient clinic staff to easily and quickly assess frailty for the purposes of diagnosing frailty, screening patients for clinical trials and quantifying the impact of interventions on frailty status [11].

There are over 25 subjective and objective frailty assessment methods [12]. Two methods that focus on physical frailty are the Cardiovascular Health Study (CHS) frailty phenotype method (Fried’s phenotype method) [3], which is the most widely cited method [13] and the Short Performance Physical Battery (SPPB) [14]. These tools are extensively published on for the assessment of frailty [3, 15–18], however, the components of the assessments differ, which may have implications on the feasibility of incorporating these assessments into clinical practice. The Fried phenotype method considers weight loss, exhaustion, physical activity level, grip strength, and walking speed, whereas the SPPB is a test of walking speed, balance and ability to complete chair stands. Although the Fried phenotype method predominantly assesses physical frailty, it has good construct validity [3], convergent validity [19], concurrent validity [20] and predictive validity [21] for assessing frailty. Moreover, the tool has been shown to be sensitive to change following an intervention in frail patients [9]. The SPPB has been identified as one of the best physical performance tests to identify frail adults [18]. It is one of the primary outcomes of interest in large, multicomponent trials in older adults [22]. Like the Fried phenotype method, the SPPB has good concurrent validity when compared to other measures of frailty [15, 16], internal consistency [23], and satisfactory short (1 week) and long-term (6 month) reliability [24, 25].

The primary aim of this study was to determine the prevalence of frailty using the two methods, and agreement between the methods. The secondary aim was to determine the feasibility of assessing frailty at a geriatric out-patient clinic.

## Methods

### Design, setting and participants

This cross-sectional study included 120 patients from a geriatric out-patient clinic at the Centre for Healthy Aging at St. Peter’s Hospital, Hamilton Health Sciences in Hamilton, Ontario, Canada. Patients were referred to the out-patient clinic by their family physician for assessment by an interdisciplinary team, including a geriatrician, registered practical nurse and case-manager (either a registered nurse or occupational therapist). The most common reasons for referral were falls, cognitive impairment, medication review, and failure to cope in the community. All new patients attending the clinic between June to December 2013 (7 months) and July to August 2014 (2 months) were approached by a research assistant to participate in the study. All patients attending the clinic were consecutively recruited during these two recruitment windows. As we were interested in assessing frailty among all patients attending the clinic, there was no age restriction, and if patients did not speak English, but had a translator present, they were invited to participate in the study. Patients were excluded if: 1) they were non-English speaking and did not have a translator present; 2) a member of the interdisciplinary team deemed the patient unfit to participate due to cognitive impairment; 3) the patient or legally authorized representative did not provide informed consent. Participants in a wheel chair were invited to participate, as we were interested in capturing the feasibility of assessing frailty among all patients attending the clinic. Written consent was obtained from the participants or legally authorized representative prior to enrollment. The Hamilton Integrated Research Ethics Board (HiREB) approved the study.

### Frailty assessment

A research assistant was responsible for assessing frailty using: 1) Fried’s phenotype method; and 2) SPPB.

- *Fried’s phenotype method*
  Fried’s phenotype method classifies older adults as frail, pre-frail or non-frail based on five criteria [3]. For each of the criteria, the participant was classified as frail or not frail, using the following cut-offs: 1) Weight loss: more than 10 lbs. lost unintentionally in the last year; 2) Exhaustion: participants stating that they felt that everything they did was an effort or that they could not get going (from the CES-D Depression Scale) a moderate amount of the time or most of the time; 3) Physical activity (Minnesota Leisure Time Activity Questionnaire): energy expenditure <383 kcal per week for men and <270 kcal per week for women; 4) Walk time (15-ft walk): ≥ 7 sec (men height ≤ 173 cm, women height ≤ 159 cm) or ≥ 6 sec (men height > 173 cm, women height > 159 cm); 5) Grip strength (Jamar Dynamometer, Layfayette Instruments, USA) (average of three trials): ≤ 29–32 kg for men (stratified by BMI classifications) and ≤ 17–21 kg for women (stratified by BMI classifications) [3]. If the participant was unable to answer any questions due to memory problems, the accompanying legally authorized representative provided an answer, which is the approach used in other studies [26]. Participants were instructed to use an assistive ambulatory aid for the walk test if an aid was used in their normal routine. Frail participants scored below the cut-offs for three or more criteria, pre-frail participants scored below the cut-offs for one or two criteria, and non-frail participants did not score below the cut-offs for any criteria [3].
- *Short Performance Physical Battery (SPPB)*
  The SPPB consists of three assessments: 1) repeated chair stands; 2) balance tests (side-by-side, semi-tandem and tandem balance tests); 3) an eight-foot walk test [23]. Similar to Fried’s phenotype method, the participant’s scores on each component of the battery were compared to normative data and a score between zero and four was determined for each component. If participants were unable to complete a component of the test, a score of zero was given for that component. A final summary performance score out of 12 is calculated, with higher scores indicating superior lower extremity function [23]. Regarding the threshold score for frailty, community-dwelling older adults who score ≤ nine on the SPPB are most likely to be classified as frail [16] and are at risk of losing the ability to walk 400 m [27] (predictive validity). An SPPB score of ≤9 has the most desirable sensitivity (92%), specificity (80%) and greatest area under the curve (AUC =0.81) for identifying frail adults [15]. In order to classify participants as frail, pre-frail and non-frail, the following cut-offs were used: SPPB zero–six (frail), SPPB seven–nine (pre-frail), SPPB 10–12 (non-frail) [28].

### Assessment of feasibility

Various aspects of feasibility can be studied, including feasibility of the study processes, resources, management, and scientific basis [29]. Our primary outcome for feasibility was the percent of eligible and enrolled participants agreeing to the frailty assessments, with the criterion for success being ≥90%. We selected a high threshold for feasibility with the intention of conducting a prospective study in the future with this study population. Attrition rates range between 10 and 30% over nine to 12 month studies involving frail older adults [30, 31], therefore obtaining baseline frailty data on ≥90% of study participants is desirable. We also documented the resources required and the occurrence of adverse events as a result of conducting the assessments.

### Additional data collection

Body mass index (BMI) was calculated based on weight, measured in kilograms (to the nearest 0.1 kg) using a standard scale or wheel-chair scale, and height, measured in centimeters using a stadiometer. Charts were abstracted to obtain the participant’s highest level of education, diagnosis of other diseases, use of ambulatory aids, living arrangement, number of prescribed medications and self-reported number of falls in the last month. The Standardized Mini-Mental State Exam (SMMSE) score was also abstracted, and cognitive impairment was defined as SMMSE <24 [32].

### Statistical analysis

Frequencies were calculated to determine the prevalence of participants who were frail, pre-frail or non-frail. To test the agreement between measures, Cohen kappa statistics and standard errors are reported for the frail and pre-frail classifications [33]. Interpretation of kappa values was based on the suggestions by Viera and Garrett [34]. All statistical analyses were completed with SPSS (Version 22, IBM Corp.) and a *p*-value of <0.05 is considered significant.

## Results

**Fig. 1:**
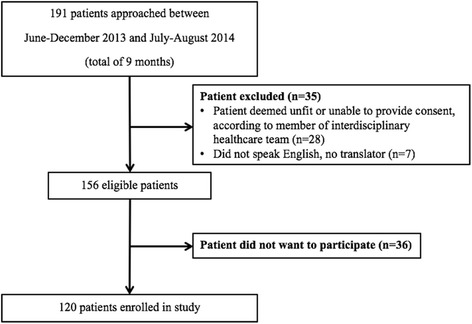
Participant recruitment flow diagram

Overall, 120 of 156 (77%) eligible patients consented to participate (Fig. 1). Participant descriptive characteristics are presented in Table 1. The mean (SD) age and BMI of participants was 80.6 (6.3) years and 26.9 (4.9) kg/m^2^, respectively. Eight patients (7%) recalled at least one fall in the past month. The mean (SD) SMMSE score was 22.7 (5.4), and approximately half (62/120) of the study participants were classified as having cognitive impairment.

**Table 1:** Descriptive characteristics of study participants (*n* = 120)

Descriptive variable	
Age, years	80.6 (6.3)
Female, n (%)	64 (53)
BMI, kg/m^2^	26.9 (4.9)
Highest level of education	
Elementary school, n (%)	29 (24)
High school, n (%)	43 (36)
Post-secondary (college or university), n (%)	28 (23)
Incomplete or other, n (%)	20 (17)
Diagnosis of other diseases	
Hypertension, n (%)	76 (63)
Myocardial infarction, n (%)	5 (4)
Type 2 diabetes, n (%)	27 (22)
Osteoarthritis, n (%)	51 (42)
Osteoporosis, n (%)	32 (27)
Type of ambulatory aid used	
Cane (single or 4-point), n (%)	22 (18)
Rollator walker, n (%)	22 (18)
Wheelchair, n (%)	3 (2)
Living arrangement	
House, n (%)	57 (47)
Apartment, n (%)	30 (25)
Retirement home, n (%)	16 (13)
Incomplete or missing, n (%)	17 (14)
Number of patients who experienced a fall in the last month, n (%)	8 (7)
Number prescribed medications	9 (4)
Cognitive impairment (SMMSE <24), n (%)	62 (52)

Data are expressed as mean (standard deviation), unless otherwise specified

*Abbreviations*: *BMI* body mass index, *SMMSE* standardized mini-mental state exam

### Prevalence of frailty and agreement between assessment methods

According to Fried’s phenotype method, 39/110 (35%) patients were frail, 63/110 (57%) patients were pre-frail and 8/110 (7%) were non-frail. According to the SPPB, 55/109 (50%) patients were frail, 38/109 (35%) patients were pre-frail and 16/109 (15%) patients were non-frail. There was fair to moderate agreement between methods for determining which participants were frail (0.488 [0.082], *p* < 0.001) and pre-frail (0.272 [0.084], *p* = 0.002). Cognitive impairment (SMMSE <24) was common among the participants who were deemed frail, pre-frail and non-frail (Table 2).

**Table 2:** Prevalence of cognitive impairment (SMMSE <24) based on frailty status

	Prevalence of cognitive impairment based on Fried’s phenotype method n (%)	Prevalence of cognitive impairment based on SPPB n (%)
Frail	23/39 (59%)	31/55 (56%)
Pre-frail	32/62 (52%)	19/38 (50%)
Non-frail	6/9 (67%)	8/16 (50%)

Cut-offs used: Fried’s phenotype method: Frail- 3 or more criteria, Pre-frail- 1–2 criteria, Non-frail- 0 criteria. SPPB: Frail- ≤6, Pre-frail- 7–9, Non-frail- 10–12

*Abbreviation*: *SPPB* Short Performance Physical Battery

### Feasibility assessment

The feasibility data are presented in Table 3. Of the 120 participants enrolled, 110 participants (92%) and 109 participants (91%) agreed to attempt Fried’s phenotype method and SPPB, respectively. Of the 21 patients or legally authorized representatives who declined the frailty assessments, 13 patients or legally authorized representatives (62%) declined because they believed that the assessment would increase the amount of time that the clinic visit would take (Table 3). The resources required to complete both assessments were the same, with the exception of a grip strength dynamometer being required for Fried’s phenotype method and a chair being required for the SPPB. No adverse events were reported for either assessment.

**Table 3:** Feasibility measures for assessing frailty

	1. Percent of participants agreeing to attempt assessment^a^	2. Resources required	3. Safety
Fried’s phenotype method	Agree to attempt = 110/120 (92%) Decline = 10/120 (8%) Reasons for decline: • Lack of time to complete assessment, *n* = 7 • Patient wheel-chair bound, *n* = 3	1) Research Assistant 2) Data collection sheets 3) Floor tape for 15-ft walk course 4) Stop watch 5) Grip strength dynamometer	0 adverse events
Short Performance Physical Battery (SPPB)	Agree to attempt = 109/120 (91%) Decline = 11/120 (9%) Reasons for decline: • Lack of time to complete assessment, *n* = 6 • Fear of falling, *n* = 2 • Patient wheel-chair bound, *n* = 3	1) Research Assistant 2) Data collection sheets 3) Floor tape for 8-ft walk course 4) Stop watch 5) Chair	0 adverse events

^a^Primary feasibility outcome. Criteria for success: ≥ 90% agreeing to attempt assessments

## Discussion

In this cohort of older adults at a geriatric out-patient clinic, the prevalence of frailty was 35% according to Fried’s phenotype method, and 50% according to the SPPB. There was fair to moderate agreement between assessment methods. At least 90% of enrolled participants agreed to be assessed for frailty, which suggests that according to our criteria for feasibility, it is feasible to assess frailty in a geriatric out-patient clinic.

This study showed that most patients attending the out-patient clinic were frail or pre-frail. Cognitive impairment was also evident in approximately 50% of our participants, which may contribute to the development of frailty [35]. However, we found that cognitive impairment was equally common in the pre-frail and non-frail groups. We were unable to discern the directionality of the relationship between cognitive impairment and frailty, given the small sample size and cross-sectional design, and this should be explored in a larger study. Similar to our findings, Tavassoli and colleagues found that 39% and 54% of older patients attending a geriatric clinic were pre-frail or frail, respectively, according to Fried’s phenotype method [8]. In addition, 75% of the participants had an SPPB score ≤ nine [8]. Kim and colleagues found that among older men attending a geriatric out-patient clinic, 34% were considered to be frail and 50% were considered to be pre-frail according to Fried’s phenotype method [36]. The high prevalence of frailty and pre-frailty in our study indicates a need to intervene to prevent further health declines and disability. An intervention similar to that described by Cameron and colleagues would be ideal, as components of physical frailty (i.e.*,* gait speed, physical activity level) and SPPB scores improved after 12 months of a tailored, multifaceted intervention based on baseline frailty characteristics [31].

When identifying frail participants, there was fair to moderate agreement between methods used. These findings are similar to those reported by Theou et al. and Islam et al. who both showed agreement between Fried’s phenotype method and other frailty assessment methods (i.e.*,* Clinical Frailty Scale) [17, 37]. While the relationship between SPPB and frailty has been reported [15], this is the first study to examine the agreement between the SPPB and other frailty assessment tools, using the SPPB cut-offs for frail and pre-frail. Given that physical activity and gait speed are important indicators of frailty [38], it is not surprising that agreement between the SPPB and Fried’s phenotype method is fair to moderate. Our findings show that for identifying frail or pre-frail older adults, either method could be used, but consideration should be given to other aspects of feasibility.

In a clinical setting similar to our study, Kim and colleagues explored the feasibility of assessing frailty using Fried’s phenotype method in 162 male veterans attending a geriatric clinic [36]. While no criterion for feasibility was stated, the authors concluded that it is feasible to assess frailty using Fried’s phenotype method, but that the assessment took approximately 15-20 min [36]. Another recent study revealed that Fried’s phenotype method takes 10 min or less to administer [39]. One of the more time consuming components of Fried’s phenotype method may be the Minnesota Leisure Time Activity Questionnaire. Fried’s phenotype method has been modified over 200 times, with the physical activity and weight loss components being modified most often [40]. Eckel and colleagues developed a modified six-item physical activity questionnaire, based on the Minnesota Leisure Time Activity Questionnaire, and found that scores obtained from the modified version were predictive of scores obtained with the original questionnaire [41]. We also question the validity of this questionnaire for a geriatric out-patient population, as many participants received a score of frail on this component of the assessment because an energy expenditure of zero kilocalories per week was recorded. It may be that the activities captured on the questionnaire are not appropriate for an older geriatric out-patient clinic population, and another method of assessing activity level, such as the Physical Activity Scale for the Elderly (PASE) may be more appropriate [42]. In another feasibility study, Maxwell and colleagues aimed to determine the feasibility of assessing frailty in hospitalized older adults in 5 min or less using the Vulnerable Elders Survey (VES-13), Barthel Index and Life Space Assessment questionnaires [43]. The main barrier to participant enrollment was the absence of a surrogate respondent to provide consent and assist with the questionnaires [43]. Also, like our study, a research assistant was responsible for completing the assessments, which resulted in a high completion rate. However, having a dedicated person responsible for assessing frailty is not sustainable in most healthcare settings, therefore, future studies should investigate the feasibility of clinic staff completing the assessments. Given that we found that agreement between methods is fair to moderate and there were no adverse events reported for either method, clinical staff should consider the added time that administering the Fried phenotype method may take [36], the differences in equipment requirements (i.e.*,* grip strength dynamometer), and appropriateness of the physical activity questionnaire that is part of the Fried phenotype method. Clinicians may also want to consider other contributors to frailty, such as weight loss and exhaustion, which aren’t directly assessed using the SPPB. In addition to considering aspects of feasibility, Norman and Streiner suggest that in judging the appropriateness of a tool for a clinical setting, the validity (concurrent, predictive, convergent, content validity) and reliability (internal consistency and stability) of the assessment method should be considered [44]. Many of these features are also highlighted as key criteria for frailty assessment tools, with the addition of a tool’s ability to predict patient response to therapy, be supported by biologic mechanisms, be feasibly applied, and to align with the purpose (i.e., risk prediction) of assessing frailty [1, 13, 39].

There are various strengths to this study. We objectively assessed feasibility using criteria set a priori*,* and we assessed frailty using two characteristically different, yet valid and reliable methods. The Fried’s phenotype method that was used was not modified from the original method, which is rare as Theou and colleagues reported that only 24 of 264 studies assessed frailty using the original method [40]. We also investigated the agreement between the assessment methods based on published cut-offs for frailty and pre-frailty, whereas other studies have reported the agreement for the frailty category only, or have not included the SPPB in agreement analyses.

There are also study limitations to acknowledge. This study used a small sample of participants from one geriatric out-patient clinic and all assessments were performed by a research assistant. We would have preferred to have clinic staff complete the frailty assessments and use additional geriatric clinic sites. These factors would improve the generalizability of our results to other clinic settings that don’t have access to additional resources, such as a research assistant. However, by involving a research assistant, we were able to assess frailty on over 90% of study participants who met the study criteria. The high prevalence of frailty and pre-frailty in this population provides rationale to involve clinic staff in assessing frailty in the future. The study was also conducted between 2013 and 2014, and occurred intermittently over 9 months. While we don’t believe that the feasibility results would differ if recruitment occurred contiguously over the study period, the prevalence of frailty may be underestimated as lower activity levels, exhaustion, weight loss and falls may be more common during the winter months. In addition, the frailty assessment tools that were selected do not specifically assess cognitive and social aspects of health, which may also contribute to frailty. Finally, members of the interdisciplinary team excluded patients from the study if they deemed the participant unfit to participate, which included some patients with severe cognitive impairment and/or no legally authorized representative present at the appointment to provide consent. Therefore, we may have excluded some of the most frail patients.

## Conclusion

Assessing frailty using Fried’s phenotype method or the SPPB is feasible in a geriatric out-patient clinic, based on the high rate of eligible participants who agreed to the frailty assessments. Knowing that eligible study participants are willing to undergo these frailty assessments is crucial for starting a randomized controlled trial targeting frailty in this population. The high prevalence of frailty and pre-frailty in this population indicates a need for continued frailty assessment and tailored interventions to prevent the progression of frailty. Given that resources should be used and allocated wisely in clinical settings, implementing new measures requires consideration about feasibility, the purpose of using the new measure and the value-added by implementing the measure. Frailty data seems to provide added value for predicting incident disability, beyond age, sex and number of comorbidities, particularly for adults over the age of 80 years [45], making the geriatric out-patient clinic an ideal setting for frailty assessment. As a result of this study, frailty is now being assessed in the clinic, and the results of the assessments are incorporated into case-management planning. In addition, the results have helped tailor interventions to include fall prevention and exercise programs to prevent further declines in frailty. Other clinicians and researchers aiming to assess frailty in clinical practice will want to consider various aspects of feasibility of each assessment before directing resources toward implementing the assessment method.
